# Characterization of fine specificity of the immune response to a *Plasmodium falciparum* rhoptry neck protein, PfAARP

**DOI:** 10.1186/s12936-016-1510-4

**Published:** 2016-09-07

**Authors:** Aakanksha Kalra, Paushali Mukherjee, Virander S. Chauhan

**Affiliations:** Malaria Research Group, International Centre for Genetic Engineering and Biotechnology, Aruna Asaf Ali Marg, New Delhi, India

**Keywords:** Malaria, Humoral and cellular response, Peptides, Immunodominant regions/epitopes

## Abstract

**Background:**

Immunological characterization of potential blood-stage malaria antigens would be a valuable strategy in the development of an effective vaccine. Identifying B and CD4^+^ T cell epitopes will be important in understanding the nature of immune response. A previous study has shown that *Plasmodium falciparum* apical asparagine-rich protein (PfAARP) stimulates immune response and induces potent invasion-inhibitory antibodies. Antibodies to PfAARP provide synergistic effects in inhibition of parasite invasion when used in combination with antibodies to other antigens. In the present study, an attempt was made to identify B cell and CD4^+^ T cell epitopes of PfAARP.

**Methods:**

Balb/c mice were immunized with recombinant PfAARP and both cellular and humoral responses were analysed at various time points. Computerized databases [immune epitope database (IEDB) and B cell epitope prediction (BCEPred)] were used to predict epitope sequences within PfAARP and predicted peptides were synthesized. In addition, nine 18 amino acid, long-overlapping peptides spanning the entire length of PfAARP were synthesized. Using these peptides, B cell and CD4^+^ T cell responses in PfAARP immunized mice were measured by ELISA and ELISPOT assays.

**Results:**

Here, it is demonstrated that immunization of mice with PfAARP induced long-lasting, high-titre antibodies (4 months post immunization). Also, the recombinant protein was effective in inducing a pronounced Th1 type of immune response quantified by IFN-γ ELISA and ELISPOT. It was found that the predicted peptides did not represent the immunogenic regions of PfAARP. However, of the nine overlapping peptides, three peptides (peptides 3, 5 and 7) were strongly recognized by PfAARP-immunized sera and represented B cell epitopes. Also, peptide 3 elicited IFN- γ response, suggesting it to be a T-cell epitope.

**Conclusions:**

Induction of long-lasting humoral and cellular response on PfAARP immunization in mice underscores its possible use as a blood-stage malaria vaccine candidate. Mapping of immunogenic regions may help in designing fusion chimera containing immunologically relevant regions of other vaccine target antigens and/or for multi-component vaccine candidates.

## Background

In the search for an effective vaccine against *Plasmodium falciparum*, the deadliest form of malaria, a plethora of proteins from all parasite stages have been investigated over the past several decades. It is during the blood-stage of infection that malaria disease occurs and, therefore, special attention has been given to merozoite surface proteins and invasion ligands as targets for novel vaccines and therapeutics [1–3]. Blood-stage vaccines aim to control the severity of the disease and ultimately the clearance of blood-stage parasites. This would prevent any symptoms of malaria and additionally block transmission [4]. A successful malaria vaccine will need to target a large population of antigenically diverse malaria parasites to protect people against the blood-stage infection [5, 6].

Protective immunity to blood-stage malaria is elicited through complex interactions between both humoral and cell-mediated responses [7]. It is well established that B cells and antibodies play a crucial role in immunity to malaria. Naturally acquired immunity in individuals living in malaria-endemic areas, which is slow to develop, is dependent largely on the acquisition of a repertoire of specific, protective antibodies directed against the blood-stage antigens [8]. Antibodies are associated with controlling levels of parasitaemia by directly inhibiting merozoite invasion of erythrocytes or opsonizing merozoites for phagocytosis. In addition to the high-titre, long-lasting humoral response and memory response, development of an effective cellular response is equally important for an effective vaccine. The T cell response to an antigen is dependent on both the cytokine environment and antigen persistence during *Plasmodium* infection. Studies in both mice and humans have shown that pro-inflammatory cytokines, IFN-γ, TNF and IL-12, essentially mediate protective immunity to erythrocytic-stage malaria parasites [9]. *Plasmodium*-specific interferon-gamma (IFN- γ) responses in vitro are associated with both human experimental and natural infections (reviewed in [10]). CD4^+^ T cells have been shown to control infection through IFN-γ production and provide help for the B-cell response required for control and elimination of infected red blood cells (RBCs) [11, 12]. In general, IFN-γ from CD4^+^ T-cells has been shown to be important in maintaining strain-transcending blood-stage immunity against *Plasmodium chabaudi* infection [13]. Similarly, in humans, IFN-γ contributes to a vast network of protective responses against blood-stage parasite and correlated with better anti-parasite immunity [11, 14].

In addition to the type of immune response generated, determination of B and T cell epitopes in context of malaria vaccine development has been a useful exercise [15–18]. Identification of immunodominant regions may be helpful in the design of fusion chimera and/or for multi-component vaccine candidates [16]. Determination of short/specific regions may also present the advantage of large-scale production of chimeric peptides, more stable than recombinant proteins, comprising multiple malarial epitopes, at low cost. Advances in the in silico B and T epitope prediction databases have further assisted research interests towards epitope determination from potential vaccine candidates.

*Plasmodium falciparum* apical asparagine-rich protein (PfAARP) is a potential target antigen for inclusion into a malaria vaccine [19]. PfAARP is expressed in late schizont stage of the parasite and localized in the rhoptry neck [19]. *Plasmodium falciparum* apical asparagine-rich protein (PfAARP) contains an N-terminal signal sequence, asparagine repeats, a conserved polyproline stretch and a C-terminal transmembrane domain. A previous study showed that PfAARP ectodomain (amino acid 20–107) binds to human RBCs in neuraminidase and trypsin dependent manner [19]. Antibodies targeting PfAARP ectodomain (hereafter referred to as PfAARP) were effective in inhibiting parasite invasion in vitro alone and also provided synergistic effects in parasite invasion inhibition in combination with antibodies to other parasite proteins [19]. PfAARP was recognized by human serum samples from malaria-endemic regions pointing to its role in naturally acquired immunity [19]. In this study, PfAARP-specific humoral and cellular responses were analysed, and using a series of overlapping synthetic peptides, it was attempted to map the B and T cell epitopes of PfAARP in a murine model. Results in this study show that PfAARP induced high-titre, long-lived antibodies and robust cellular recall responses. Using a series of synthetic peptides, three B cell epitopes and one CD4^+^ T cell epitope of PfAARP were identified. These findings may provide rationale for designing multiple epitope sub-unit vaccine based on PfAARP and other well-known malaria vaccine candidates.

## Methods

### Expression, purification and characterization of recombinant PfAARP

PfAARP (amino acid 20–107 with a C-terminal His tag) was cloned from the full length synthetic gene construct with 5′-CACATCATCAcatatgATTCTGCGTAATAATAAAAGCC-3′ and 5′-TATATActcgagTCAGTGGTGGTGGTGGTGGTGATCTTCATTGTCTTCTTCATC-3′ as forward and reverse primers, respectively, between *Nde*I and *Xho*I restriction sites. The recombinant plasmid was transformed in *Escherichia coli* BLR (DE3) cells and the transformed cells were grown in LB broth containing kanamycin (50 µg/ml) at 37 °C until it reached an optical density of 0.6–0.8 at 600 nm (OD_600_). The expression of the recombinant protein was induced by 1 mM isopropyl-β-d-thiogalactopyranoside (IPTG) for 4 h and the expression level was analysed in un-induced and induced samples by SDS-PAGE and Western blotting.

For purification of the recombinant protein cell pellet from shake flask culture was homogenized in lysis buffer (20 mM NaH_2_PO_4_ pH 8.0, 300 mM NaCl, 5 mM benzamidine-HCl, 10 mM imidazole and 100 µg/ml lysozyme) and lysed by sonication on ice for 20 min with a 9-s pulse on/off. The lysed culture was centrifuged and the resulting clear supernatant was loaded on to equilibrated Ni^2+^ charged, streamline-chelating resin for immobilized metal affinity chromatography (equilibration buffer: 20 mM NaH_2_PO_4_ pH 8.0, 300 mM NaCl). The resin was subsequently washed with five column volumes of equilibration buffer. The bound protein was eluted with a step gradient of imidazole (10 to 500 mM) in 20 mM NaH_2_PO_4_ and 10 mM NaCl, pH 8.0. Eluted fractions were analysed by SDS-PAGE and protein-containing fractions were pooled and loaded onto equilibrated Q-Sepharose column with equilibration buffer (20 mM NaH_2_PO_4_ and 10 mM NaCl) for further purification. The protein was eluted with a step gradient of NaCl (10 to 500 mM) in 20 mM NaH_2_PO_4_ pH 7.0. The eluates containing purified PfAARP were pooled and protein concentration was determined by bicinchoninic acid assay (BCA).

Homogeneity of the purified protein was assessed by SDS-PAGE, by Western blot analysis with anti-His antibody and by reverse phase chromatography (RP-HPLC) on an analytical C_18_ column (Discovery Supelco). Endotoxin content of the purified protein was estimated by Limulus Amoebocyte Lysate (LAL) gel clot assay (Charles River Endosafe).

### Immunization of mice with PfAARP formulated with Freund’s adjuvant

Balb/c mice were bred in the animal housing facility of International Centre for Genetic Engineering and Biotechnology (ICGEB) under pathogen-free conditions as per recommendation of the guide for the care and use of laboratory animals (ICGEB, India). ICGEB is licensed to conduct animal studies for research purposes under the registration number 18/1999/CPCSEA (dated 10/1/99). All the experimental protocols were approved by the ICGEB Institutional Animal Care and Use Committee (IAEC: MAL).

For longevity study and B cell epitope mapping, a group of five mice (6–8 weeks old female mice) were immunized subcutaneously in the hindfoot pads with 25 µg of PfAARP in complete Freund’s adjuvant and subsequently boosted with the same amount of protein in incomplete Freund’s adjuvant. Three booster doses were given on day 15, 30 and 65 post priming and sera was isolated on day 45, 60, 81, 102, 132, 162, and 192 post prime immunization. Antibody titres were followed until day 192 viz 4 months after the last booster immunization. In parallel, a group of five mice was immunized with PBS formulated with the adjuvant to serve as control mice.

For cellular response, phenotypic characterization and T cell epitope mapping, two groups of three mice each were immunized with 25 µg of the recombinant antigen in complete Freund’s adjuvant and given a booster dose in incomplete Freund’s adjuvant with same amount of antigen on day 21 before harvesting spleen. Post-booster immunization, one group of mice was harvested on day 35 and another on day 42. Mice injected with adjuvant-formulated PBS were used as the control group. Both the control and the test group mice were sacrificed on the same day and isolated splenic cells were analysed for T cell response.

### Enzyme-linked immunosorbent assay (ELISA)

Antibody responses in mice towards specific antigen and peptides were evaluated by ELISA. Briefly, 96-well micro-titre plates (Costar, Corning Inc.) were coated with recombinant PfAARP (200 ng per well) or peptides (800 ng per well) in 0.06 M carbonate–bicarbonate buffer (pH 9.6) and incubated overnight at 4 °C. Antigen-coated plates were washed three times with PBS-T (phosphate buffer saline-Tween 20) and blocked with 2 % skimmed milk in PBS (pH 7.2) for 2 h at 37 °C. Antigen-coated plates were sequentially incubated with serial dilutions of the immune sera from mice and then with HRP-conjugated (horse-radish peroxidase) rabbit anti-mouse secondary antibody in diluent (0.25 % skimmed milk in PBS-T) for 1 h each at 37 °C. The plates were washed with PBS-T and the enzyme reaction was developed by a mixture of *o*-phenylenediaminedihydrochloride (OPD) and hydrogen peroxide (H_2_O_2_) in citrate phosphate buffer (pH 5.0), the reaction was stopped by 2 N H_2_SO_4_ and OD was recorded at 492 nm by a Versamax ELISA reader (Molecular Devices). Cut-off values were determined as the mean plus twice the standard deviation (SD) for the pre-immunization sera and were used to determine the endpoint titres.

For competitive ELISA, day 45 anti-PfAARP sera was pre-incubated with three different concentrations (25, 50, 100 µg/ml) of PfAARP, peptide 1, peptide 3, peptide 5 and peptide 7 for 30 min at room temperature (RT). Reactivity of these sera was tested against PfAARP by ELISA as per protocol described above.

### Cell preparation

PfAARP-immunized mice were sacrificed at respective time points (day 35 and 42) and spleen was harvested. Spleens were dispersed into single cell suspensions by grinding using blunt ends of a 5-ml syringe and centrifuged to collect dispersed spleen cells. After centrifugation, erythrocyte lysis was performed by adding 1 ml of RBC lysis buffer [10 mM KHCO_3_, 155 mM NH_4_Cl and 0.1 mM EDTA (pH 7.4)] for 1 min and stopped with 9 ml of PBS. cells were centrifuged at 250×*g* for 10 min, resuspended in 10 ml complete medium (RPMI 1640 supplemented with 10 % FBS, 50 μg/ml gentamicin and 2 mM l-glutamine) (cRPMI), passed through a 70-µm cell strainer to get single cell suspension and counted with haemocytometer.

### Flow cytometry

Phenotypic characterization of B and CD4^+^ T cells in splenic cell population of PfAARP immunized mice was done by flow cytometry of total spleen cells. B cell characterization was done using B220, GL7, CD38 and IgG1 labelled antibodies and CD4^+^ T cells were characterized using CD4, CD44, CD11a, and CD62L labelled antibodies. Briefly, spleen cells (1 × 10^6^ cells) from day 42-sacrificed mice were stained with respective labelled antibodies (BD Biosciences). Cells were stained for 30 min at 4 °C in the dark, washed twice with FACS buffer (PBS, 2 % FBS and 1 g/l sodium azide) and then fixed with 1 % paraformaldehyde (PFA) in PBS for 30 min on ice. Stained cells were acquired with a FACS Calibur fluorescence-activated cell sorter (FACS) machine (BD Biosciences) and further analysed by FlowJo software (Tree Star). Profiles are presented as 5 % probability contours with outliers. Percent values with SD and fold change observed for PBS (control) and PfAARP (test) immunized mice were presented.

### Cytokine response

Splenic cell cultures and cytokine assays were performed at the specified time points to measure secreted IFN-γ response. Splenic cells were resuspended in cRPMI and plated at 5 × 10^5^ cells/well in 96-well, flat-bottom plates (Costar, Corning Inc.). The spleen cells were stimulated in vitro at 37 °C in 5 % CO_2_ with PfAARP at 50 μg/ml or with the different peptides (both the predicted and overlapping) (50 μg/ml) or with medium alone for 48 h. Concanavalin A (Con A) at a concentration of 1 μg/ml was used in all experiments as positive control. Supernatants were taken after 48 h of stimulation in culture and tested by sandwich ELISA for interferon gamma (IFN-γ) with BD-IFN-γ ELISA commercial kit as per manufacturer’s protocols.

### IFN-γ ELISPOT assay

IFN-γ ELISPOTs (BD Bioscience) were conducted according to the manufacturer’s instructions. Briefly, 96-well ELISPOT plates (Millipore Multiscreen-HA) were coated with capture IFN-γ antibody (1:1000) overnight at 4 °C to detect IFN-γ expressing cells. After three to five washes with sterile PBS, plates were blocked by cRPMI for 2 h at 37 °C. Spleen cells from PfAARP-immunized mice were then added to the wells at 5 × 10^5^ cells per well in the presence of recombinant protein or respective peptides at concentration of 50 µg/ml, in final volume of 200 µl and incubated for 48 h at 37 °C in a 5 % CO_2_ incubator. A negative control containing 5 × 10^5^ cells per well without any peptide or protein and a positive control containing 5 × 10^5^ cells per well in the presence of 1 µg/ml Con A were tested under the same conditions. After washing, plates were incubated with biotinylated anti-IFN-γ detection antibodies for 2 h at RT followed by streptavidin-HRP conjugate for 45 min at RT. After washing three times with PBS-T and once with PBS, 100 μl of soluble HRP substrate (3-amino-9-ethylcarbazole substrate in 0.05 M acetate buffer, pH 5.5) was added to each well and incubated at RT for 30 min and then the plates were rinsed with water. Wells were scanned and spots were counted by computer-assisted ELISPOT image analysis CTL Analyzer and software (CTL Analyzers). Images of individual wells of the ELISPOT plates were analysed for cytokine spots by comparing wells containing antigen-stimulated immune cells and control wells with unstimulated immune cells. Cut-off values were determined as the mean values plus twice the SD values obtained for control wells with unstimulated cells. Thus, values which were greater than the cut-off values were treated as positive ELISPOT responses.

### Prediction of B and T cell epitopes in AARP

B and T cell epitopes were predicted in mouse using bio-informatic databases. The databases used to predict B cell epitopes were immune epitope data base (IEDB) and B-cell epitope prediction (BCEPred). The prediction tools used in IEDB were Chou and Fasman beta turn prediction, Parker hydrophilicity prediction and Bepipred linear epitope prediction [20] whereas in BCEPred hydrophilicity, flexibility, accessibility, turns, exposed surface, polarity, and antigenic propensity were simultaneously used for prediction [21].

Fifteen amino acid, long helper T cell epitopes were predicted from IEDB for H2-IAb, H2-IAd and H2-IEd alleles of the mouse locus H2-I with ‘IEDB recommended prediction method’ using the Consensus approach [22]. An alternate prediction method in IEDB ‘SMM-align’ was also used to predict the direct MHC binding affinity of the peptides for the three alleles of H2-I locus.

### Peptide synthesis

The two predicted peptide sequences, Peptide A (^32^P I S K T N E EEE G K I N I N^47^) and Peptide B (^92^E S D N D E E E E E D E E D N E D L^107^) from PfAARP were synthesized by standard, solid-phase synthetic methods [23]. Briefly, peptides were synthesized on Wang Resin (0.44 mmol/g) using Fmoc methodology at 0.3 mM scale. Couplings were performed by using *N*,*N*-diisopropylcarbodiimide and Fmoc deprotection was performed with 20 % piperidine in dimethylformamide. After addition of the final residue, the resin was rinsed with dimethylformamide/dichloromethane/methanol and dried. The final peptide de-protection and cleavage from the resin was achieved with 20 ml of trifluoroacetic acid/phenol/water/triisopropylsilane (17.6:1:1:0.4) for 2 h. The crude peptide was precipitated with cold ether, filtered, lyophilized, and stored at −20 °C as dry powder. Peptide purity was confirmed by RP-HPLC using water acetonitrile gradient on a C-18 column (Discovery Supelco).

Eighteen amino acid long nine overlapping peptides spanning PfAARP were commercially synthesized by standard solid phase synthesis methods (GenicBio Limited, China).

### Statistical analysis

Statistical analyses were performed using both Excel (Microsoft Office) and GraphPad Prism statistical software (GraphPad Software Inc.). Data are expressed as the mean ± SD when derived from three or more values. To determine the level of significance, a Student t test was performed and a p < 0.05 was considered significant.

## Results

### Purification and characterization of PfAARP

**Fig. 1:**
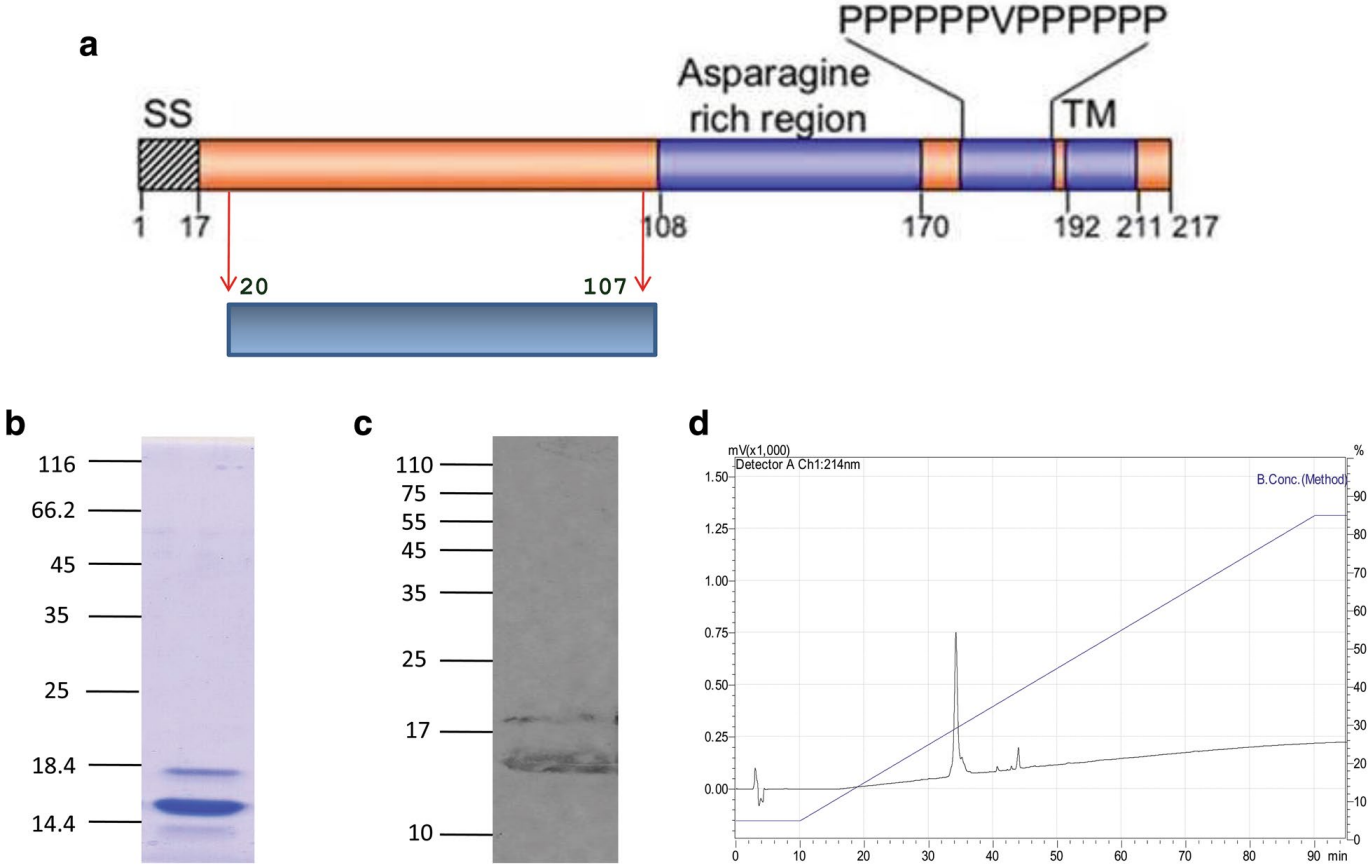
Purification and characterization of PfAARP. **a** Position of PfAARP ectodomain in the protein sequence. **b** Mobility of recombinant purified protein on SDS-PAGE. **c** Western blot of recombinant purified protein with anti-His monoclonal antibody. **d** RP-HPLC profile of purified protein showing the purity of the protein

PfAARP described in this study comprises amino acid 20–107, devoid of signal sequence, asparagine repeats, polyproline stretch, and transmembrane domain of the protein (Fig. 1a). PfAARP was expressed as a soluble protein in cytosolic fractions and purified to homogeneity by a three-step chromatographic procedure, including immobilized metal affinity chromatography followed by anion exchange chromatography and finally RP-HPLC, to remove endotoxin from the purified protein. Purified PfAARP showed an apparent mobility at ~17 kDa on SDS-PAGE instead of its predicted molecular mass of 11.6 kDa (Fig. 1b). This discrepancy in mobility of the recombinant protein may be due to highly acidic pI (5.06) of the protein and the unusual amino acid composition. Such aberrant migration on SDS-PAGE has been observed with a number of other malaria proteins, such as RESA and also for *Toxoplasma* proteins (another Apicomplexan parasite) [24, 25]. Identity of the recombinant protein was confirmed by Western blot analysis under reducing conditions with anti-His monoclonal antibody (Fig. 1c). The results from both SDS-PAGE analysis and RP-HPLC showed that the purity of the recombinant protein was greater than 95 % (Fig. 1d). Gel clot assay based on LAL reagent kit showed that endotoxin level of the IEX purified fraction was high (~5000 EU per 25 µg protein) which was significantly reduced to ~2.5 EU per 25 µg of the protein on RP-HPLC of IEX fractions. This endotoxin level is well below the permissible limit (30 EU per 25 µg protein) for bacterially expressed proteins used for immunization studies in small animals. Application of RP-HPLC for endotoxin removal from bacterially expressed proteins has been used previously for other proteins, such as human cryptic plasminogen-derived domain Kringle 5 [26]. Purified PfAARP with low, acceptable level of endotoxin content was used for immunization in mice.

### Humoral responses generated to PfAARP

**Fig. 2:**
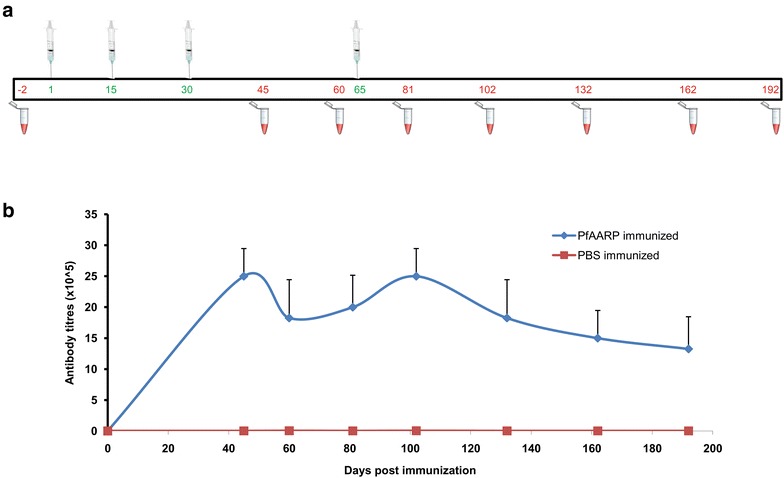
Humoral response generated to PfAARP. **a** Immunization schedule stating prime, booster doses and bleed of mice for humoral response. **b** Kinetics of humoral response generated to the protein in terms of endpoint titres

To study immunogenicity of PfAARP, a group of five mice were immunized and boosted with protein formulated with Freund’s adjuvant, and serum was isolated at different time points as described in the immunization schedule (Fig. 2a). The kinetics of humoral response by ELISA showed that high-titre antibodies (of the order of 10^6^) were raised upon PfAARP immunization and the response persisted until day 192, showing that PfAARP-specific antibodies stayed in circulation even 4 months after the last booster dose (Fig. 2b). Since the half-life of secreted IgGs is not more than 21 days, it may be inferred from these results, that longevity of the observed antibody response may be the result of both long-lived plasma cells (LLPCs) and/or memory B cells (MBCs) [27]. As expected, the control mice did not show any antigen-specific antibody response to PfAARP (Fig. 2b).

In order to understand the phenotypic and functional changes involved in generation of long-lived humoral response, changes in surface markers of mice splenic B cells induced by PfAARP immunization were analysed (Table 1). Flow cytometric results showed a ~1.4-fold up-regulation in the expression of CD38 on B220^+^ splenic cells in protein-immunized mice as compared to PBS-immunized mice. This increase in CD38 expression indicated B cell activation and isotype switching of immunoglobulins. Isotype switching in splenic cells of PfAARP-immunized mice was further confirmed by a ~1.8-fold increase in expression of IgG1 on B220^+^ splenic cells. This increase in isotype switching and IgG1^+^ cells correlated well with PfAARP-specific high antibody titres observed in the serum. In addition to B cell activation and isotype switching, immunization with PfAARP also induced germinal centre (GC) formation as observed by a ~2.5 increase in B220^+^ GL7^+^ cells in spleen, the site for generation of MBCs. GC formation in spleen of protein immunized mice was further supported by ~five-fold increase in the expression of CD27 on B220^+^ splenic B cells. An increase in CD27 on B cells promoted the formation of GC and consequent IgG production [28]. Together, these results clearly indicated that upon AARP immunization, there was an early and persistent B cell activation, induction of GC formation, as well as IgG^+^ MBC generation.

**Table 1:** Phenotypic characterization of splenic CD4^+^ T cells and B cell in PfAARP and PBS immunized mice

Cell markers	Adjuvant control	Protein immunized	Fold change
B220^+^GL7^+^	0.34 ± 0.05	0.89 ± 0.49	2.50
B220^+^CD38^+^	24.5 ± 3.53	33.95 ± 7.70	1.38
B220^+^CD27^+^	0.6 ± 0.45	3.40 ± 2.18	5.67
B220^+^IgG1^+^	0.31 ± 0.13	0.55 ± 0.09	1.80
CD4^+^CD44^+^	22.35 ± 2.89	25.8 ± 0.84	1.15
CD4^+^CD11a^+^	9.68 ± 2.85	18.05 ± 1.9	1.86
CD4^+^CD62L^+^	28.45 ± 11.8	16.9 ± 0.56	0.59

Spleen cells from recombinant protein (test) and PBS (control) immunized mice were stained with respective labelled antibodies, acquired by FACS Calibur and analysed by FlowJo. Percent values with standard deviation and the fold change observed for the control and test mice are presented

### Cellular response generated to PfAARP

**Fig. 3:**
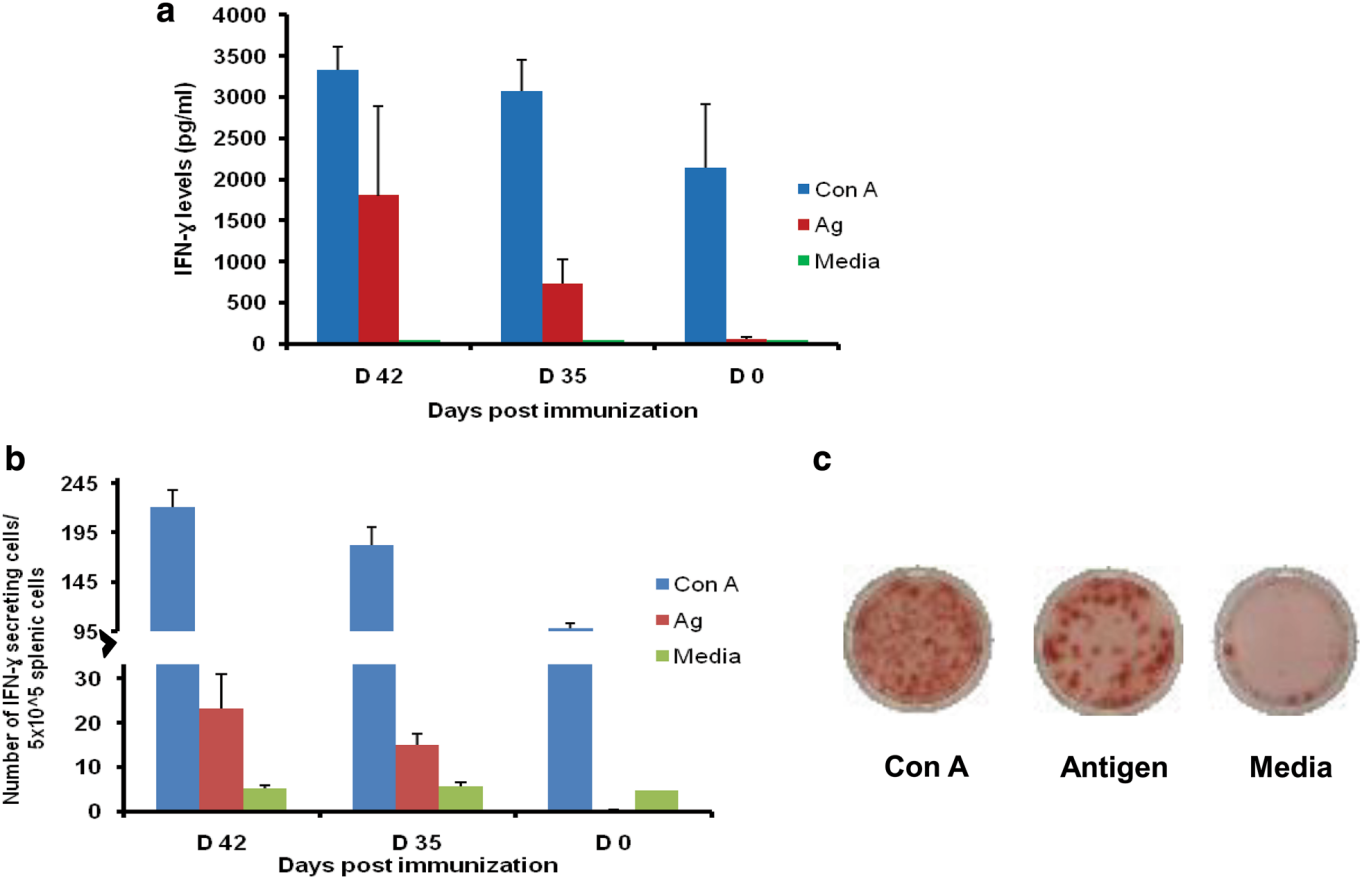
Cellular response generated to PfAARP. **a** Secretion of IFN-γ by splenic cells and **b** Number of IFN-γ secreting cells when splenic cells from mice immunized with either PfAARP or PBS were stimulated with protein or media (negative control) at different time points. Con A was used as positive control. **c** Representative images of ELISPOT wells stimulated with Con A, PfAARP and media

To analyse cell-mediated immune (CMI) responses, spleen cells were harvested from immunized mice (PfAARP immunized) and control mice (PBS immunized) at different time points and cultured in medium alone or stimulated with PfAARP. CMI responses induced by PfAARP were kinetically analysed by IFN-γ-ELISA and ELISPOT. Spleen cells from control mice stimulated with AARP produced insignificant level of IFN-γ throughout the course of the experiment (~57.4 pg/ml for day 42) (Fig. 3a). On the other hand, as expected, spleen cells from PfAARP-immunized mice produced high IFN-γ (~738 pg/ml) on day 35 and the level increased markedly by day 42 (~1804 pg/ml) post prime immunization (p < 0.05) (Fig. 3a). This increase in secreted IFN- γ levels in splenic cells from protein-immunized mice stimulated with PfAARP may be due to the formation of antigen specific Th1 cells upon protein immunization. Results from ELISPOT assay also showed a significant number of IFN-γ-producing Th1 cells in PfAARP-immunized mice as compared to PBS-immunized mice [~1.5 (day 0) vs ~15.5 (day 35) and ~29.677 (day 42)] (Fig. 3b). Con A was used as a positive control for both IFN-γ ELISA and ELISPOT assays. Figure 3c showed representative images of ELISPOT wells stimulated with Con A, PfAARP and media. These results, therefore, indicated that an effective cellular, particularly, IFN-γ response, was generated during PfAARP immunization. IFN-γ has been shown to be a critical cytokine produced during malaria infection that controls parasite growth, probably by splenic macrophage activation to phagocytose-parasitized RBCs [10]. Studies have also shown that there may exist a correlation of pRBC (extracellular merozoites and intra-erythrocytic parasites) specific IFN-γ to protection (reviewed in [11]).

To analyse the cell surface expression of activation markers in PfAARP- and PBS-immunized mice, cells were stained with anti-CD4, -CD44 and -CD11a, markers which increase on activation of T cells, and -CD62L, a molecule with a decreased cell surface expression on T cell activation (Table 1). Flow cytometric results showed ~1.15-fold and ~1.86-fold up-regulation in CD44 and CD11a expression, respectively, on gated CD4^+^ T cells in AARP-immunized mice compared to PBS-immunized mice. Also, there was a significant reduction (~0.59-fold change) in the expression of CD62L on gated CD4^+^ T cells in PfAARP-immunized mice. These results clearly indicated that following immunization, PfAARP induced an activated phenotype on CD4^+^ T cells and a strong Th1 response. Also, it has been shown that cognate T cell responses significantly contribute to the generation of potentially effective antibody response in both infection-induced and vaccine-induced immunity against malaria [29]. Strong T cell response induced by PfAARP is in quite contrast to a number of crucial parasite antigens, such as PfMSP-1_19_ that show weak T cell recognition, which compromises their efficacies as vaccine candidates [30].

### Prediction of epitopes, peptide synthesis and immunogenicity testing of predicted epitopes

**Fig. 4:**
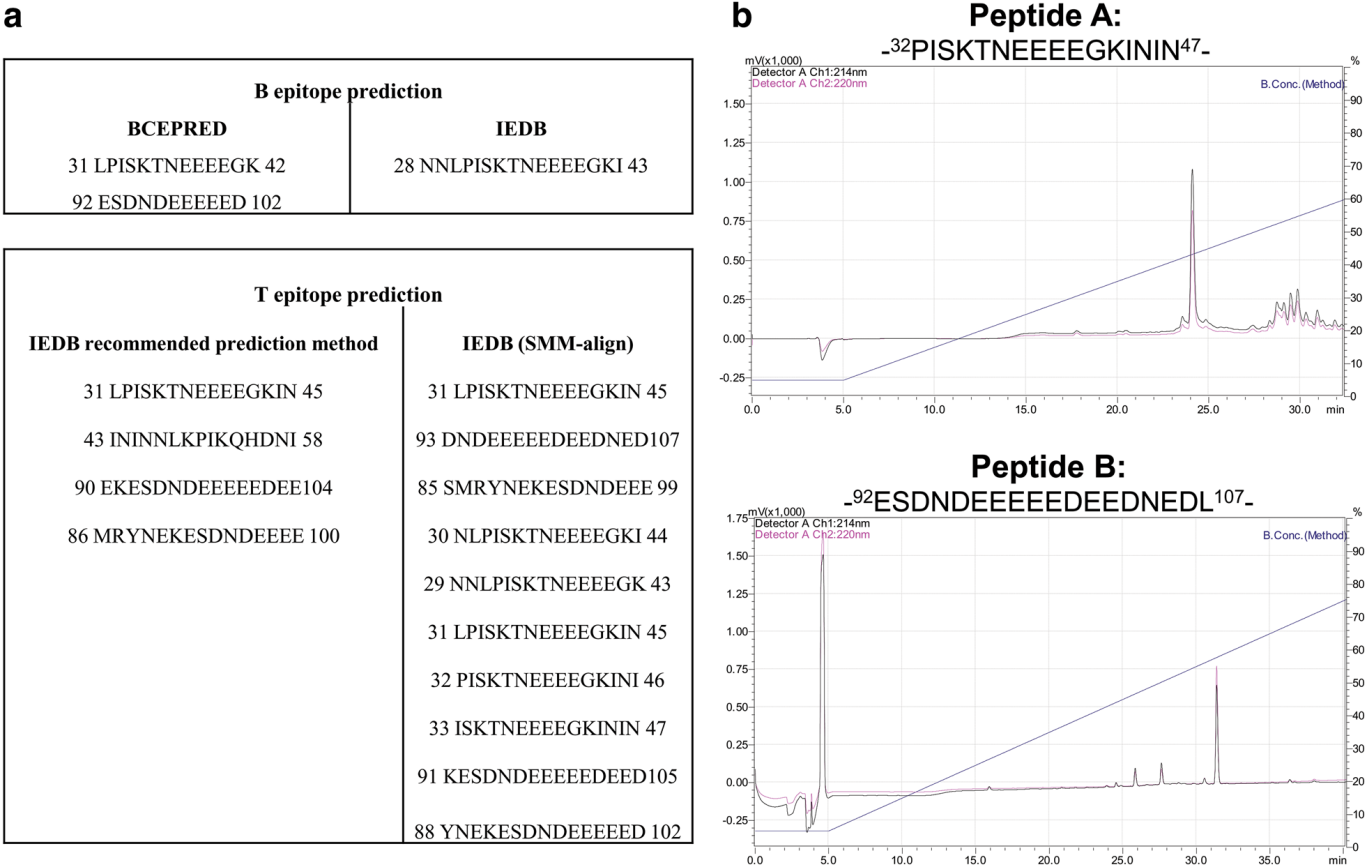
Epitope prediction and peptide synthesis. **a** B and T cell epitopes were predicted by IEDB and BCEPred. **b** Selected peptides were synthesized using Fmoc methodology and their purity was determined by RP-HPLC

Since a robust humoral and cellular immune response is generated in mice immunized with PfAARP, it was next attempted to determine linear minimal immunogenic regions of the protein. Two B cell epitopes (10–15 residues) were predicted by BCEPred and one epitope (16 residues) by IEBD (Fig. 4a). Also, ten helper T cell epitopes (15 residues) were predicted by IEDB-recommended prediction method and four helper T cell epitopes by SMM-align method to the three alleles of H-2I MHC locus of mice (Fig. 4a). Peptides predicted as B and T cell epitopes mainly overlapped in two fragments of PfAARP, of which one was localized in the centre and other in C-terminus of the protein. Therefore, two peptides corresponding to residue 32–47 and 92–107 were synthesized (Peptide A and B) by standard solid phase methods [23]. Peptide purity was analysed by RP-HPLC and was shown to be >90 % (Fig. 4b).

**Fig. 5:**
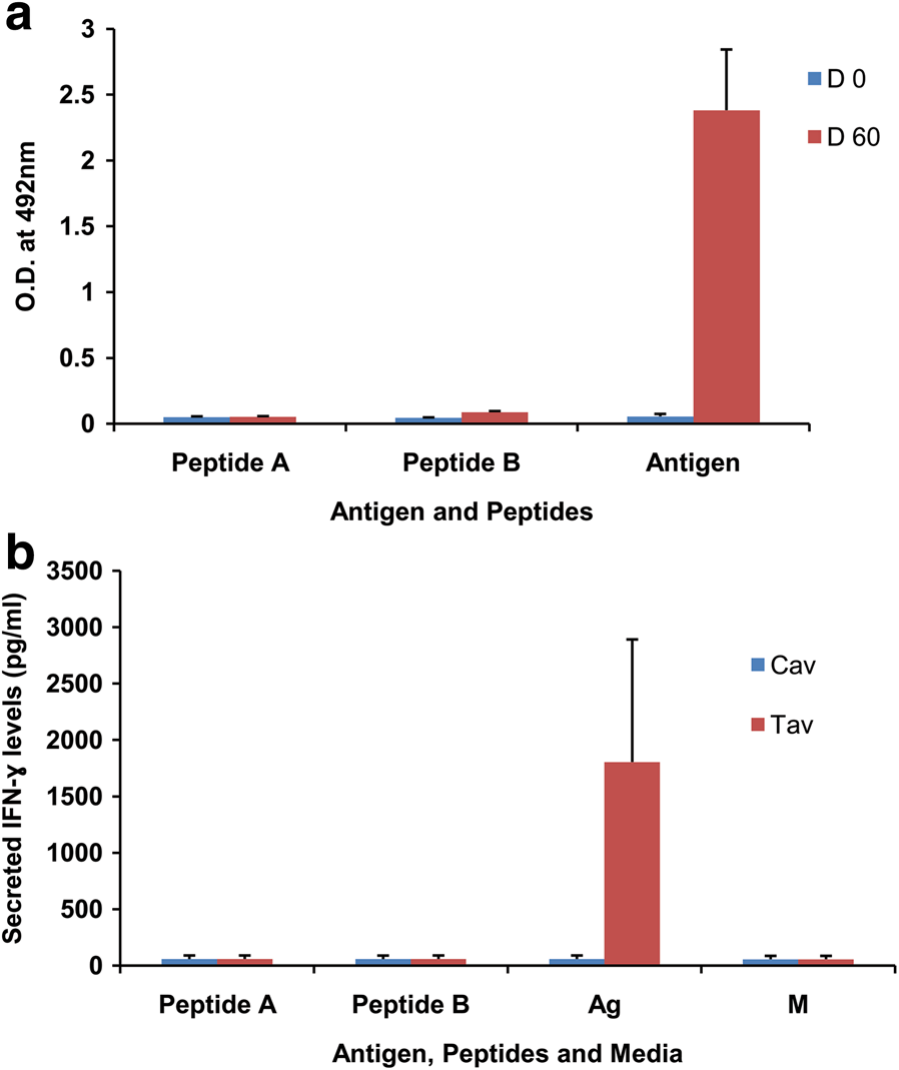
Evaluation of peptides A and B as epitopes. **a** ELISA was performed for the two peptides with protein-immunized sera to evaluate as B cell epitopes; ELISA with recombinant antigen and with pre-immune sera was used as positive and negative controls, respectively. **b** Splenic cells from protein immunized mice were stimulated with peptides and IFN-γ secretion in the culture supernatant was analysed by ELISA to evaluate T cell epitopes; stimulation with protein and media were used as positive and negative control

PfAARP-immunized mice sera were tested against Peptides A and B by ELISA to determine whether they represented B cell epitopes. While, immune sera at 1:500 dilution showed high reactivity to the protein (OD_492_ ~2.5), there was no reactivity with Peptide A (OD_492_ ~0.05) or B (OD_492_ ~0.08) (Fig. 5a). These results suggested that the predicted peptides in fact do not represent B cell epitopes. As expected, pre-immune sera did not show reactivity with PfAARP or Peptide A and B.

Peptides A and B were also analysed in a cellular response assay to determine whether these represented putative CD4^+^ T cell epitopes. Following in vitro stimulation of splenic cells from PfAARP-immunized mice with the recombinant protein, splenic cells produced significantly higher levels of IFN-γ (~1800 pg/ml) when compared to control mice (~57 pg/ml) (Fig. 5b). However, both Peptide A and B failed to stimulate IFN-γ production (~50 pg/ml) in splenic cells and thus do not represent CD4^+^ T cell epitopes. These results were somewhat surprising but at the same time clearly indicated that predicted peptides may not always turn out to be actual immunodominant epitopes. Experimental validation of computationally predicted epitopes for CelTOS, a protein expressed in motile stages of malaria parasite, indicated the limited accuracy of prediction algorithms [31]. A recent study with three novel viral-vectored antigens (PfUIS3, PfLSA1 and PfLSAP2) also supported that, of the large number of predicted epitopes only a few could be validated experimentally [32].

### Evaluation of overlapping peptides as B and T cell epitopes

**Fig. 6:**
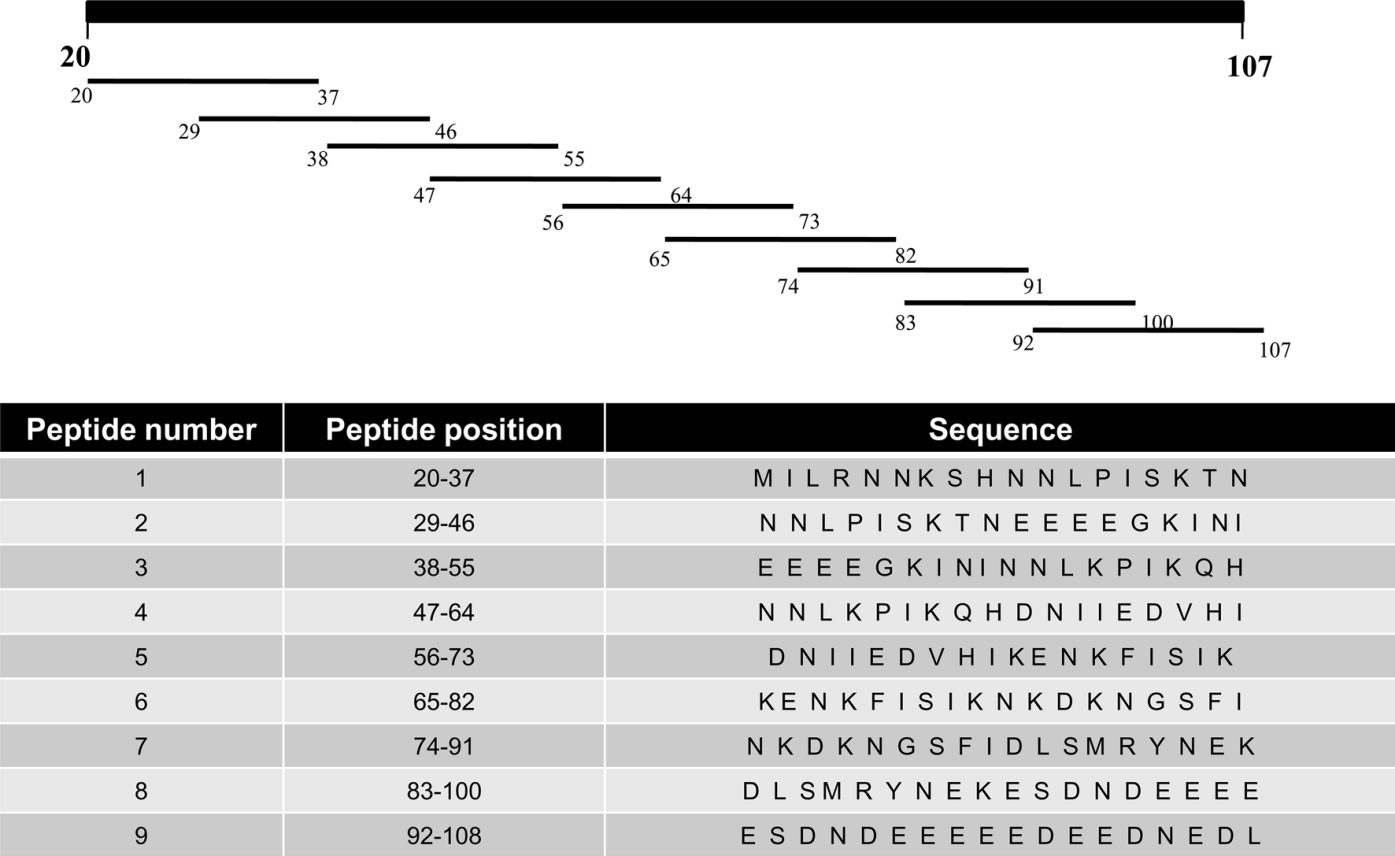
Overlapping peptides of PfAARP. Eighteen amino acid overlapping peptides were synthesized where each next peptide overlaps previous peptide by nine amino acids

Since predicted peptide sequences did not turn out to be immunodominant epitopes of PfAARP, a series of overlapping peptides covering the whole sequence of PfAARP was synthesized. These nine peptides, each 18 residues long with an overlap of nine amino acids (Fig. 6), were obtained from a commercial source (GenicBio Ltd).

**Fig. 7:**
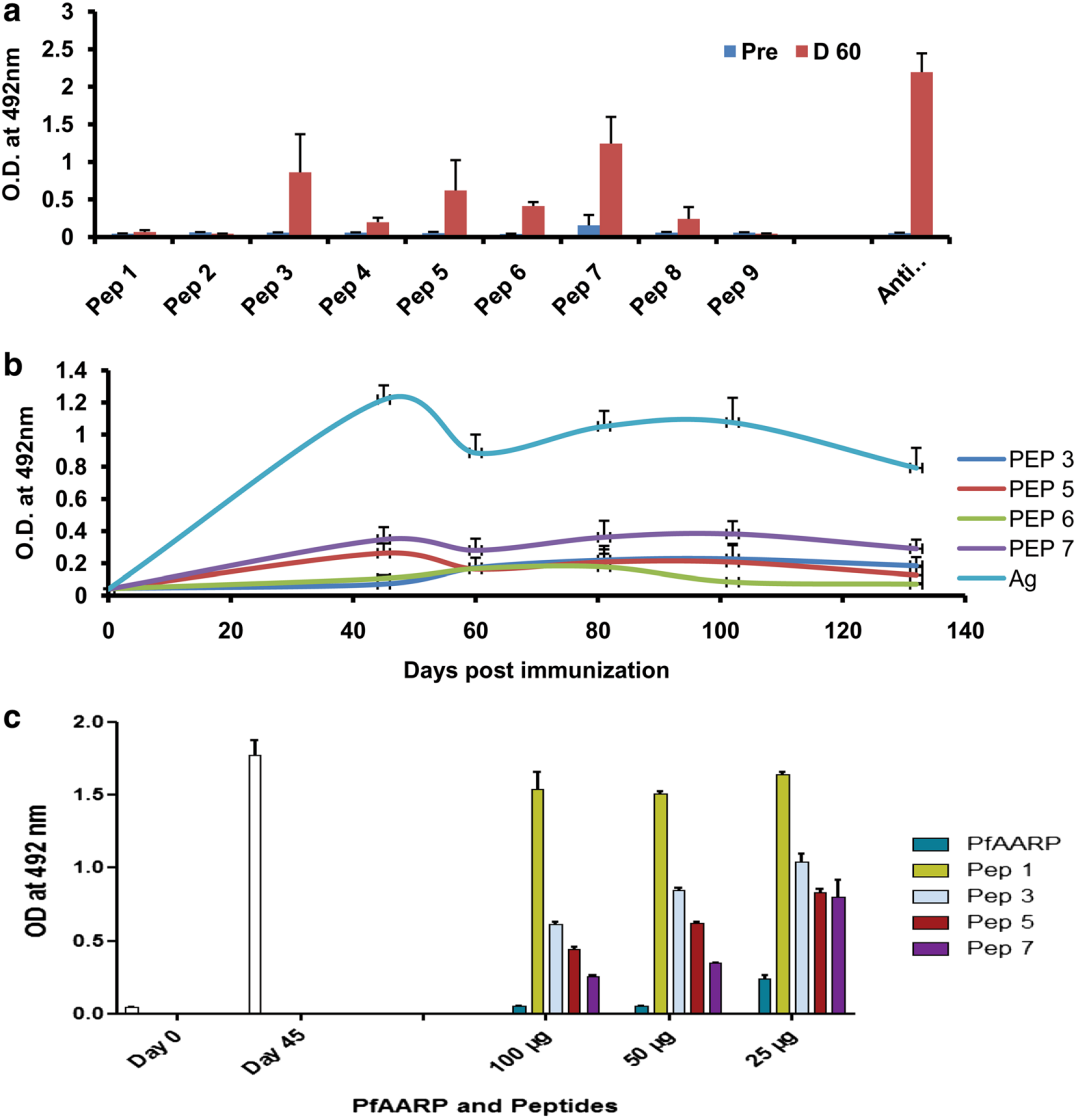
Evaluation of overlapping peptides as B cell epitopes. **a** ELISA was performed for the nine overlapping peptides with protein-immunized sera at a dilution of 1:1000. Reactivity of recombinant protein and pre-immune sera was used as positive and negative control, respectively. **b** Kinetics of the sera to peptides 3, 5, 6, and 7 with respect to recombinant protein. **c** ELISA of PfAARP antisera pre-incubated with different concentrations (25, 50 and 100 µg/ml) of PfAARP, peptide 1, peptide 3, peptide 5 and peptide 7 to PfAARP at a dilution of 1:1000. Reactivity of pre-immune sera and immune sera without pre-incubation was used as controls. White bar indicates the reactivity of day 45 anti-PfAARP sera with PfAARP

Peptides 1–9 were evaluated for their reactivity with antisera from PfAARP-immunized mice by ELISA. Reactivity of day-45 sera at a dilution of 1:1000 with PfAARP (OD_492_ ~2.1) was used as the positive control and the pre-immune sera (OD_492_ ~0.1) was used as negative control. ELISA results showed that PfAARP antisera reacted with peptides 3, 5, 6 and 7 (Fig. 7a); significant reactivity was seen with peptide 7 (OD_492_ ~1.2) followed by moderate reactivity with peptide 3 (OD_492_ ~0.8) and peptide 5 (OD_492_ ~0.6) and low but positive response to peptide 6 (OD_492_ ~0.4), other peptides did not show reactivity with the antisera. ELISA of serially diluted immune sera with these peptides showed that the endpoint titres were in the range of 10^5^ as against the 10^6^ towards PfAARP. The kinetics of immune response to PfAARP with regard to peptides 3, 5 and 7 showed a peak at day 45 and another at day 102 whereas for peptide 6, the antibody response peaked only once at day 45 (Fig. 7b). Thus, the kinetics of anti-PfAARP sera towards peptides is similar to that observed with the recombinant protein.

To further investigate the specificity of anti-PfAARP sera binding to peptides 3, 5 and 7, a competitive ELISA was performed as described previously. Anti-PfAARP serum was pre-incubated with different concentrations (25, 50 and 100 µg/ml) of PfAARP, peptide 1, peptide 3, peptide 5, and peptide 7, and then these sera were assessed for their reactivity to PfAARP. ELISA results clearly showed that on pre-incubation with PfAARP, reactivity of anti-PfAARP sera to the recombinant protein decreased in a concentration-dependent manner with ~95 % reduction at 100 µg/ml PfAARP (Fig. 7c). The reactivity of anti-PfAARP sera to PfAARP also decreased significantly on pre-incubation with synthetic peptides in a concentration-dependent manner with maximum reduction observed on pre-incubation with peptide 7 (~80 % reduction at 100 µg/ml) followed by peptide 5 (~60 % reduction at 100 µg/ml) and peptide 3 (~40 % reduction at 100 µg/ml) (Fig. 7c). However, there was no reduction in reactivity of PfAARP antisera when incubated with peptide 1, a peptide which did not react to PfAARP antisera suggesting that reactivity of peptides 3, 5 and 7 to anti-PfAARP sera is specific. These results suggested that peptides 3, 5 and 7 represented B cell epitopes of PfAARP with peptide 7 being the dominant one.

**Fig. 8:**
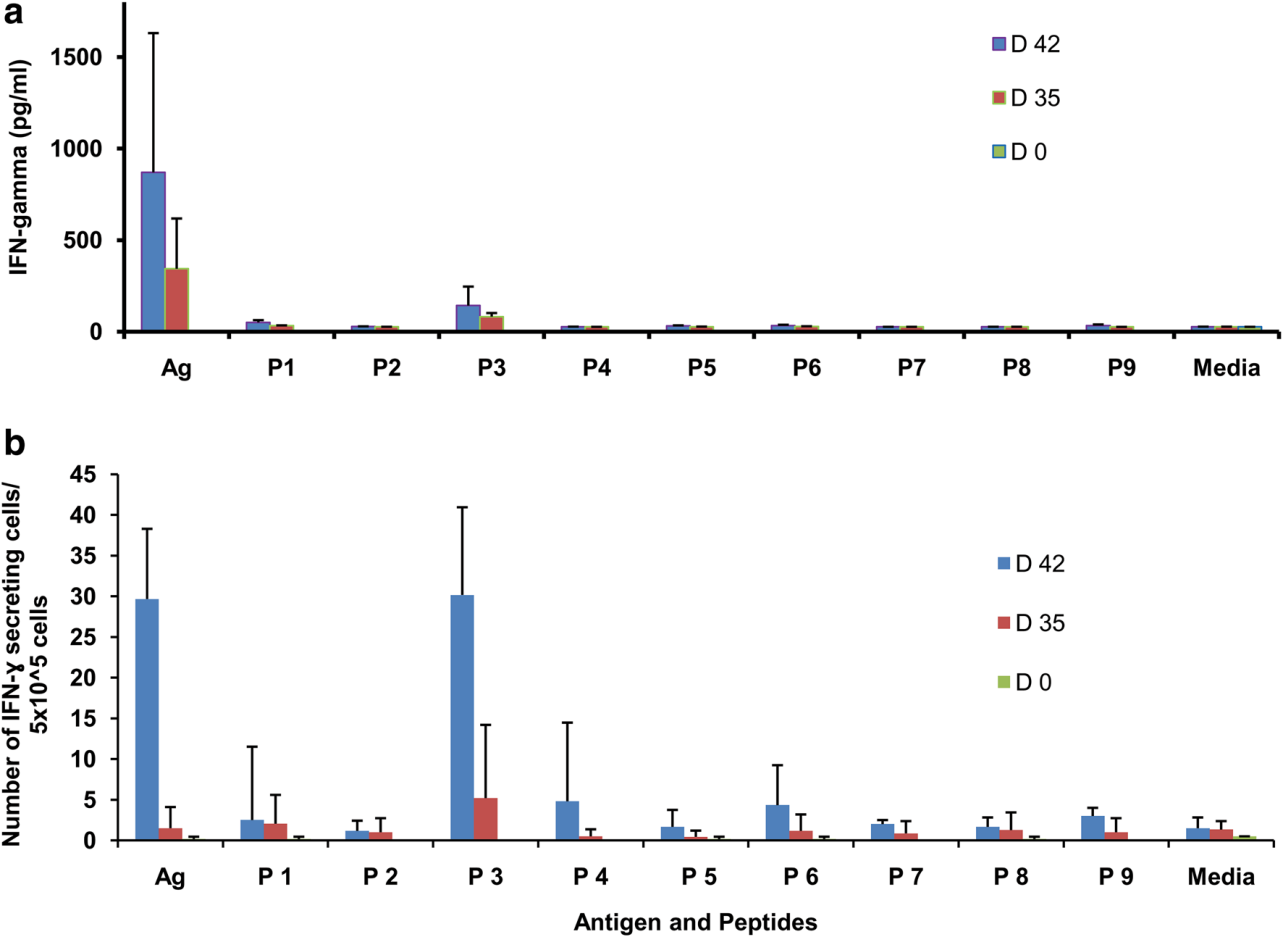
Evaluation of overlapping peptides as T cell epitopes. Assessment of IFN-γ response either released (**a**) or number of IFN-γ secreting Th1 cells (**b**) on stimulation of protein-immunized splenic cells with protein, peptides and media. Splenic cells from PBS-immunized mice (*D 0*) were used as negative (adjuvant) control

The nine overlapping peptides were also tested for the presence of T cell epitopes by IFN-γ based cellular response assay. Spleen cells from AARP-immunized mice were stimulated with nine overlapping peptides and change in IFN-γ level or number of IFN-γ-producing Th1 cells was determined by ELISA and ELISPOT, respectively. Out of the nine peptides, only peptide 3 was able to induce IFN-γ secretion in the culture supernatant. At day 35 following primary immunization, peptide 3 induced IFN-γ levels to ~200 pg/ml and by day 42 the level increased to ~535 pg/ml compared to ~57 pg/ml by splenic cells of PBS immunized mice (Fig. 8a). Splenic cells from day 35 and day 42 mice were stimulated with different peptides for 48 h to determine the numbers of IFN-γ-secreting cells. As observed from ELISPOT results, peptide 3 induced the significant proportion of IFN-γ-secreting cells ~30.166 (day 42) and ~5.82 (day 35) as against ~1.5 in the control group mice. No significant reactivity was observed with other peptides (Fig. 8b). These results indicated that peptide 3 represented T cell immunodominant epitope, found at amino acids #38–55 of PfAARP, although the computational methods were not able to predict this sequence as a putative B and/or T cell epitope.

Epitope mapping of crucial antigens has been a useful exercise that has been done using multiple methods, including monoclonal antibodies, X-ray crystallography and Nuclear magnetic resonance techniques [33–36]. For example, a peptide was identified from PfRH5 by virus-like particle (VLP) approach and a mAb to the peptide completely inhibited parasite invasion in vitro [17]. A synthetic peptide representing circularized structure of five NANP repeats of circumsporozoite protein (CSP) was developed depending on the crystal structure of CSP repeat region [37]. Although these methods are extremely reliable, they tend to be resource intensive, time consuming and have rather low throughput for fine epitope mapping. In contrast, overlapping peptides, in combination with computational methods are low cost, efficient, and can be rationally employed to identify linear B cell epitopes [15, 18, 38–40]. This approach has been used extensively to identify epitopes of some crucial parasite antigens, such as AMA-1, SERA-5, MSP-2, MSP-9, and the selected peptides have been analysed as potential vaccine candidates alone and in combination with peptides to other antigens [15, 38, 39, 41].

## Conclusion

The fine specificity of immune response generated to PfAARP, a *P. falciparum* potential blood-stage vaccine candidate was analysed in a murine model. Immunization with PfAARP induced a high-titre, long-lived antibody response which stayed in circulation for more than 4 months post immunization. Also, the recombinant protein was effective in inducing a pronounced Th1 type of immune response quantified by IFN-γ ELISA and ELISPOT. On immunization, PfAARP induced activation of B and CD4^+^ T cells, immunoglobulin isotype switching and formation of GC in splenic cell population. It was also found that the predicted epitopes in fact did not represent B and T cell epitopes of PfAARP. However, one T cell and three B cell epitopes of PfAARP were mapped by analysing overlapping peptides spanning the complete protein, the efficacy of which could be further evaluated to be a component of sub-unit vaccines. These results support the further evaluation of this vaccine candidate in a challenge model.

## Abbreviations

BCA: bicinchoninic acid assay
BCEPred: B cell epitope prediction
CMI: cell-mediated immune
Con A: concanavalin A
cRPMI: complete RPMI
CSP: circumsporozoite protein
ELISA: enzyme linked immuno sorbent assay
ELISPOT: enzyme linked immunospot assay
FACS: fluorescence-activated cell sorter
GC: germinal centre
HRP: horse-radish peroxidase
H_2_O_2_: hydrogen peroxide
ICGEB: International Centre for Genetic Engineering and Biotechnology
IEDB: immune epitope database
IFN-γ: interferon-gamma
IPTG: isopropyl-β-d-thiogalactopyranoside
LAL: limulus amoebocyte lysate
LLPC: long-lasting plasma cells
MBC: memory B cells
OD: optical density
OPD: *o*-phenylenediaminedihydrochloride
PBS: phosphate buffer saline
PfAARP: *Plasmodium falciparum* apical asparagine-rich protein
PFA: paraformaldehyde
RBC: red blood cells
RP-HPLC: reverse-phase high performance liquid chromatography
RT: room temperature
SD: standard deviation
VLP: virus-like particle
